# Elevated neuregulin-1*β* levels correlate with plasma biomarkers of cerebral injury and high stroke risk in children with sickle cell anemia

**DOI:** 10.1016/j.endmts.2021.100088

**Published:** 2021-02-14

**Authors:** Christopher Chambliss, Tatayana Richardson, John Onyekaba, Juan Cespedes, Annette Nti, Keri Oxendine Harp, Iris Buchanan-Perry, Jonathan K. Stiles, Beatrice E. Gee

**Affiliations:** a Cardiovascular Research Institute, Morehouse School of Medicine; 720 Westview Drive SW, Atlanta, GA 30310, USA; b Duke University; 2138 Campus Drive, Durham, NC, 27708, USA; c Department of Microbiology, Biochemistry, and Immunology, Morehouse School of Medicine; 720 Westview Drive SW, Atlanta, GA, 30310, USA; d Department of Pediatrics, Morehouse School of Medicine; 720 Westview Drive SW, Atlanta, GA 30310, USA; e Children’s Healthcare of Atlanta; 35 Jesse Hill Jr Drive SE, Atlanta, GA, 30303, USA; f Aflac Cancer and Blood Disorders Center; 2015 Uppergate Drive, Atlanta, GA 30322, USA; g Pediatrics Institute, Emory University School of Medicine; 2015 Uppergate Drive, Atlanta, GA, 30322, USA

**Keywords:** Neuregulin, Sickle Cell, Cerebral Injury, Stroke Risk, Anemia, Children, Hemoglobin, Plasma, Platelet Derived Growth Factor, Brain Derived Neurotropic Factor

## Abstract

Stroke, or cerebral infarction, is one of the most serious complications of sickle cell anemia (SCA) in childhood, potentially leading to impaired development and life-long physical and cognitive disabilities. About one in ten children with SCA are at risk for developing overt stroke and an additional 25% may develop silent cerebral infarcts. This is largely due to underlying cerebral injury caused by chronic cerebral ischemia and vascular insult associated with SCA. We previously identified two elevated markers of cerebral injury, plasma brain-derived neurotropic factor (BDNF) and platelet-derived growth factor (PDGF)-AA, in children with SCA and high stroke risk. The objective of this study was to investigate whether neuregulin-1*β* (NRG-1), an endogenous neuroprotective polypeptide may also be elevated in children with SCA. Neuregulin-1*β* is involved in the preservation of blood brain barrier integrity and brain microvascular cell viability and is cytoprotective in conditions of heme-induced injury and ischemia. Since elevated plasma heme and ischemia are signature characteristics of SCA, we hypothesized that NRG-1 would be elevated in children with SCA, and that NRG-1 levels would also correlate with our biomarkers of cerebral injury. Plasma NRG-1, BDNF and PDGF-AA levels were measured in children with SCA and healthy Controls. Plasma NRG-1 was found to be nearly five-fold higher in those children with SCA compared to Controls. Neuregulin-1*β* was also positively correlated with both BDNF and PDGF-AA concentrations, but was not associated with degree of anemia, suggesting that NRG-1 production may be an endogenous response to subclinical cerebral ischemia in SCA warranting further exploration.

## 1. Introduction

The most common and severe form of sickle cell disease is Hemoglobin SS (also known as sickle cell anemia, SCA), which results from homozygous inheritance of a mutation of beta globin (*β*^6 (glu -> val)^) on chromosome 11. This mutation leads to polymerization of hemoglobin molecules into rod-like structures within erythrocytes, leading to the sickled shape of red blood cells under conditions of hypoxia, intracellular dehydration, or acidosis. Individuals with SCA may experience hemolytic anemia, vaso-occlusion, tissue ischemia, and chronic vascular injury, which may lead to potentially life-threatening clinical manifestations (Belcher et al., 2014, Charrin et al., 2016, Sundd et al., 2018, Uzunova et al., 2010).

Cerebrovascular disease in children in SCA is exemplified by elevated Transcranial Doppler (TCD) velocity, cerebral artery stenosis, silent cerebral infarction, and/or overt stroke (Ohene-Frempong et al., 1998, Verduzco and Nathan, 2009). Without preventive treatment, approximately 11% of children with SCA may experience a stroke before the age of 20, with 67% having recurrence of stroke within nine months (Verduzco and Nathan, 2009, Hulbert et al., 2011). Additionally, as many as 35% of children with SCA may experience asymptomatic, or “silent” cerebral infarction, identifiable by magnetic resonance imaging (MRI) (Hulbert et al., 2011, Steen et al., 2003). Both symptomatic and asymptomatic cerebral infarctions can impair normal brain development and are associated with poor school performance and lessened cognitive functioning (Kwiatkowski et al., 2009, Schatz et al., 2001). Symptomatic stroke can lead to life-long physical disabilities.

The pathophysiology of cerebrovascular disease in SCA is not well understood. We have previously identified platelet derived growth factor type-AA (PDGF-AA) and brain derived neurotrophic factor (BDNF) as predictors of cerebral infarct and stroke risk in children with SCA (Hyacinth et al., 2014, Hyacinth et al., 2012, Lance et al., 2014). Platelet derived growth factor type-AA is an endothelial and smooth muscle cell mitogen whose production is elevated in cases of acute and prolonged vascular injury (Hyacinth et al., 2014, Majesky et al., 1990, Su et al., 2008). Brain derived neurotrophic factor is found in multiple areas of both the brain and spinal cord. Production of BDNF is elevated in conditions of prolonged or chronic cerebral ischemia and reduced cerebral blood flow, and it promotes neuronal survival via cell growth, differentiation, and maintenance (Ettrup et al., 2011, Schmidt-Kastner et al., 2001, Waterhouse et al., 2012).

Neuregulin-1*β*, our neuropeptide of interest, is a 70-kDa endogenous polypeptide growth factor that triggers signal transduction through the actions of the ErbB family of receptor tyrosine kinases. This pathway is involved in nervous system development and recovery from neuronal injury (Birchmeier and Bennett, 2016, Fazzari et al., 2010, Mei and Nave, 2014, Tokita et al., 2001, Bao et al., 2003). Neuregulin-1*β* is involved in the preservation of blood brain barrier integrity and brain microvascular endothelial cell viability in conditions of heme-induced injury, which is similar to the internal milieu in sickle cell disease caused by chronic hemolysis (Liu et al., 2012, Liu et al., 2018). Neuregulin-1*β* has also been shown to promote neuronal and astrocyte survival after induced cerebral ischemia (Tokita et al., 2001, Croslan et al., 2008, Simmons et al., 2016, Surles-Zeigler et al., 2018). In rodents, exogenous administration of NRG-1 reduces cerebral infarct size in middle cerebral artery occlusion (MCAO)-induced stroke and reduces white blood cell accumulation and inflammatory cytokine production (Simmons et al., 2016, Surles-Zeigler et al., 2018, Solomon et al., 2014, Xu et al., 2005). *In vitro*, NRG-1 reduces heme-induced endothelial cell and astrocyte apoptosis, as well as ischemia-induced neuronal apoptosis (Liu et al., 2012, Liu et al., 2018, Croslan et al., 2008, Xu et al., 2005). Our group has also demonstrated the neuroprotective role of Neuregulin-1*β* (NRG-1) in heme-induced injury in cerebral malaria (Liu et al., 2012, Liu et al., 2018, Solomon et al., 2014). Because people with SCA may experience cerebral ischemia and heme-mediated vascular injury, it is likely that NRG-1 plays a role in the brain and cerebral vasculature in SCA.

The objective of this cross-sectional study was to measure plasma NRG-1 in children with SCA and determine if NRG-1 levels are correlated with severity of hemolysis or known biomarkers of stroke risk in SCA, PDGF-AA and BDNF. We hypothesized that NRG-1, being neuroprotective, would be increased in children with SCA in response to either hemolysis or cerebral ischemia (Kassim and DeBaun, 2013, Talmage, 2008). Hemolytic anemia of SCA is characterized by low hemoglobin concentration and elevated reticulocytes, so we expected that NRG-1 would be negatively correlated with hemoglobin concentration and positively correlated with absolute reticulocyte count. Additionally, we hypothesized that NRG-1 concentration would be positively correlated with PDGF-AA and BDNF levels. We measured the plasma biomarkers in children with SCA in comparison to healthy African American children without SCA. Neuregulin-1*β* levels were then correlated to PDGF-AA and BDNF levels, as well as hematologic values.

## 2. Material and methods

### 2.1. Subjects

After IRB review and written informed consent from participants, parents, and/or legal guardians, African American children ages 3 – 21 years old were recruited from Children’s Healthcare of Atlanta Sickle Cell Clinics and Morehouse Healthcare Pediatrics Clinic. Subjects with SCA had Hemoglobin SS, no transfusions in the past three months or hydroxyurea therapy within six months, or known symptomatic stroke or silent cerebral infarction. Healthy Controls had Hemoglobin AA (normal), Hemoglobin C trait (AC), sickle cell trait (Hemoglobin AS), or beta thalassemia trait.

Exclusions included acute illness, chronic diseases other than SCA, pregnancy, and cardiovascular risk factors (hypertension, diabetes mellitus, hyperlipidemia, obese BMI, or tobacco smoking). All research was carried according to the Code of Ethics of the World Medical Association (Declaration of Helsinki). The mentioned research protocols were approved by the ethics committee of Children’s Healthcare of Atlanta (CHOA IRB Protocol #14–125].

### 2.2. Neuroimaging

All subjects with SCA underwent Transcranial Doppler (TCD) screening according to STOP study methodology (Adams et al., 1998). Subjects with SCA were not routinely screened with magnetic resonance imaging (MRI) or angiography (MRA), but were tested if they had a history of acute neurologic symptoms, focal abnormalities on physical exam, developmental delay, academic problems or changes in behavior. When performed, Brain MRI and MRA images were acquired at Children’s Healthcare of Atlanta, using 3T Trio scanners (Siemens, Erlangen, Germany) using in-house protocols for sickle cell disease stroke evaluation, including non-contrasted sagittal T1, axial T1, fluid-attenuated inversion recovery (FLAIR), T2, and diffusion-weighted images with an apparent diffusion coefficient map increased the sensitivity for detection of acute ischemia. The MRA images used a 3-dimensional time of flight technique with a short echo time and included both source and maximum intensity projection reformatted images in the anterior to posterior and right to left projections to assess the patency of the intracerebral arterial vessels.

SCA subjects were classified as having “NO Cerebral Arteriopathy” if they had no known symptomatic stroke, history of acute neurologic event, or abnormal brain MRI/MRA, and TCD time-averaged maximum meant velocities in the intracerebral vasculature were less than 170 cm/s (STOP Study definition). Subjects with SCA were designated as “WITH Cerebral Arteriopathy” if they had abnormal TCD with time-averaged maximum mean velocities in the intracerebral vasculature > 200 cm/s or brain MRA showing cerebral artery stenosis. Subjects with conditional TCD velocities (170 – 199 cm/s) were not included in this study.

### 2.3. Sample collection

Venous blood (15–18 mL) was collected from all participants, which included other research studies. Subjects with SCA had complete blood counts performed at Children’s Healthcare of Atlanta clinical laboratories. Control subjects had hemoglobin electrophoresis and red blood cell indices performed by Quest Diagnostics, which did not include white blood cell or platelet counts.

### 2.4. Immunoassays

Plasma was separated by centrifugation (10 minutes at 2552 × g) and stored frozen (−80°C) until assays were run. Growth factor levels were measured using enzyme-linked immunosorbent assay (ELISA) according to manufacturer’s protocols (ABCAM, Cambridge, UK): NRG-1 (Catalog # ab100614), samples undiluted; BDNF (ab99978) with dilution factor 50; and PDGF-AA (ab100622), with dilution factor 50. The samples were read on a microplate reader (TECAN) at 450 nm. All assays were performed in duplicate for statistical analysis.

### 2.5. Statistical analysis

Data analysis and generation of graphics were performed using Prism statistical software (GraphPad Prism 7.0, San Diego, CA, USA). Analysis of statistical difference was conducted using two-tailed Mann-Whitney tests to compare non-parametric groups and Student’s t-test to compare parametric groups, where applicable. Correlation analysis was achieved using Spearman R for non-parametric data and ranked data is used for graphical representation. All p-values resulted from two-sided statistical tests with α=0.05 with significant threshold set at p < 0.05 (denoted by *). Highly significant p-values were further denoted as follows: p < 0.01 (* *), p < 0.001 (* * *), and p < 0.0001 (* * * *). Data were reported as mean ± standard deviation or median with interquartile range (IQR) where appropriate.

## 3. Results

### 3.1. Subject characteristics

Forty samples were tested including 30 subjects with SCA and 10 Controls. All participants self-identified as African American. Twice as many females than males participated. Children with SCA tended to be significantly younger than Controls (7.8 ± 3.6 vs 11.4 ± 3.4 years), with lower weight, stature and BMI (Weight: 26.8 ± 14.7 vs 49.6 ± 16.3 kg; Height: 124.8 ± 21.9 vs 151.9 ± 16.3 cm; BMI: 16.4 ± 2.8 vs 20.7 ± 3.3 kg/m^2^; BMI percentile for age: 43.9 ± 28.4 vs 80.6 ± 8.8) (Table 1).

### 3.2. Hematologic values

Complete blood counts and hemoglobin electrophoresis for the subjects are shown in Table 2. As expected, children with SCA were anemic compared to Controls, with lower red blood cell (RBC) count (3.1 ± 0.6 vs 4.6 ± 0.3 10^12^ cells/L, p < 0.0001) and hemoglobin (Hb) concentration (8.8 ± 1.0 vs 12.6 ± 0.6 g/dl, p < 0.0001). Subjects with SCA had elevated fetal hemoglobin (Hb F) compared to Controls (14.2 ± 7.3 vs < 1.0 %, p < 0.0001). Mean corpuscle volume (MCV) was not significantly different between groups. White blood cell count (WBC), absolute reticulocyte count (ARC), and platelet (Plt) data was available for children with SCA, but not collected in Controls. Absolute reticulocyte count was elevated compared to normal reference ranges, while WBC and platelets were within normal (Blumenreich, 1990, Priya and RS, 2014, Daly, 2011).

### 3.3. Neuroimaging

Twenty-six subjects with SCA had NO cerebral arteriopathy. Three subjects had prior MRI/MRA due to clinical indications. Two of those were normal. Of the four subjects with SCA WITH cerebral arteriopathy, three had abnormal TCD velocities and one had MRA showing focal stenosis of the left middle cerebral artery.

### 3.4. Neuregulin-1β and markers of cerebral injury

**Fig. 1.**
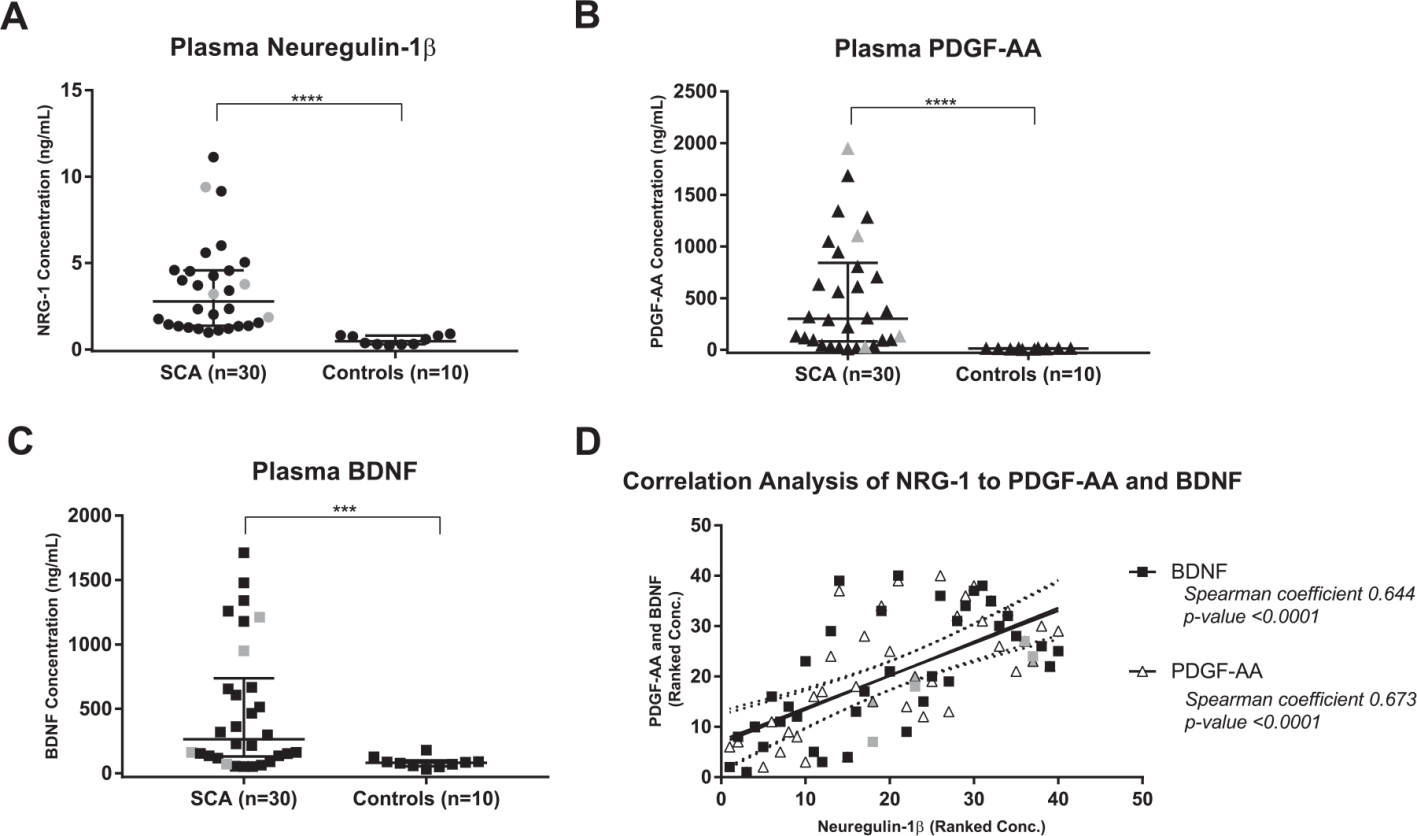
Plasma NRG-1, PDGF-AA, and BDNF concentrations in children with SCA and healthy Controls. (A) NRG-1, (B)PDGF-AA and (C) BDNF were significantly elevated in children with SCA, compared in healthy Controls. (D) Elevated plasma NRG-1 was positively corelated with elevations in either PDGF-AA or BDNF. Data points in gray represent children with SCA who have documented cerebral arteriopathy as defined by cerebral arterial stenosis or Transcranial Doppler velocity > 200cm/s. Triangles represent individuals’ ranked concentrations of plasma PDGF-AA correlated to ranked concentrations of plasma NRG-1 including a solid line of best fit and dotted 95% confidence bands. Circles represent individuals’ ranked concentrations of plasma BDNF correlated to ranked concentrations of plasma NRG-1 including a solid line of best fit and dotted 95% confidence bands. Data is reported as Median (Interquartile Range) where applicable (non-parametric data). Symbol “* * * * ” represents p-value < 0.0001 and symbol “* * * ” represents p-value < 0.001.

Neuregulin-1*β* concentrations were 5-fold higher in the children with SCA compared to Controls (median 2.774 (IQR 1.363 – 4.573) vs. 0.478 (IQR 0.293 – 0.796) ng/mL, p-value < 0.0001, Fig. 1A). Plasma PDGF-AA was 20-fold higher in children with SCA, compared to nearly undetectable levels in Controls (median 300.0 (IQR 81.0 – 841.0) vs 13.0 (IQR 8.0 – 15.0) ng/mL, p-value < 0.0001, Fig. 1B). Similarly, BDNF, was elevated 3-fold in children with SCA compared to Controls (median 263.0 (IQR 129.0 – 737.0) vs 81.0 (IQR 54.0 – 99.0) ng/mL, p-value = 0.0009, Fig. 1C). Correlation analysis between NRG-1 and the markers of cerebral injury showed a positive correlation between NRG-1 and both PDGF-AA and BDNF (Spearman r = 0.644 and 0.673 respectively, p-values both < 0.0001, Fig. 1D). Four of the children with SCA had documented cerebral arteriopathy as defined by cerebral arterial stenosis or Transcranial Doppler velocity > 200cm/s (gray symbols in Fig. 1). There was no association of cerebral arteriopathy status with biomarker levels in this small sub-group.

**Fig. 2.**
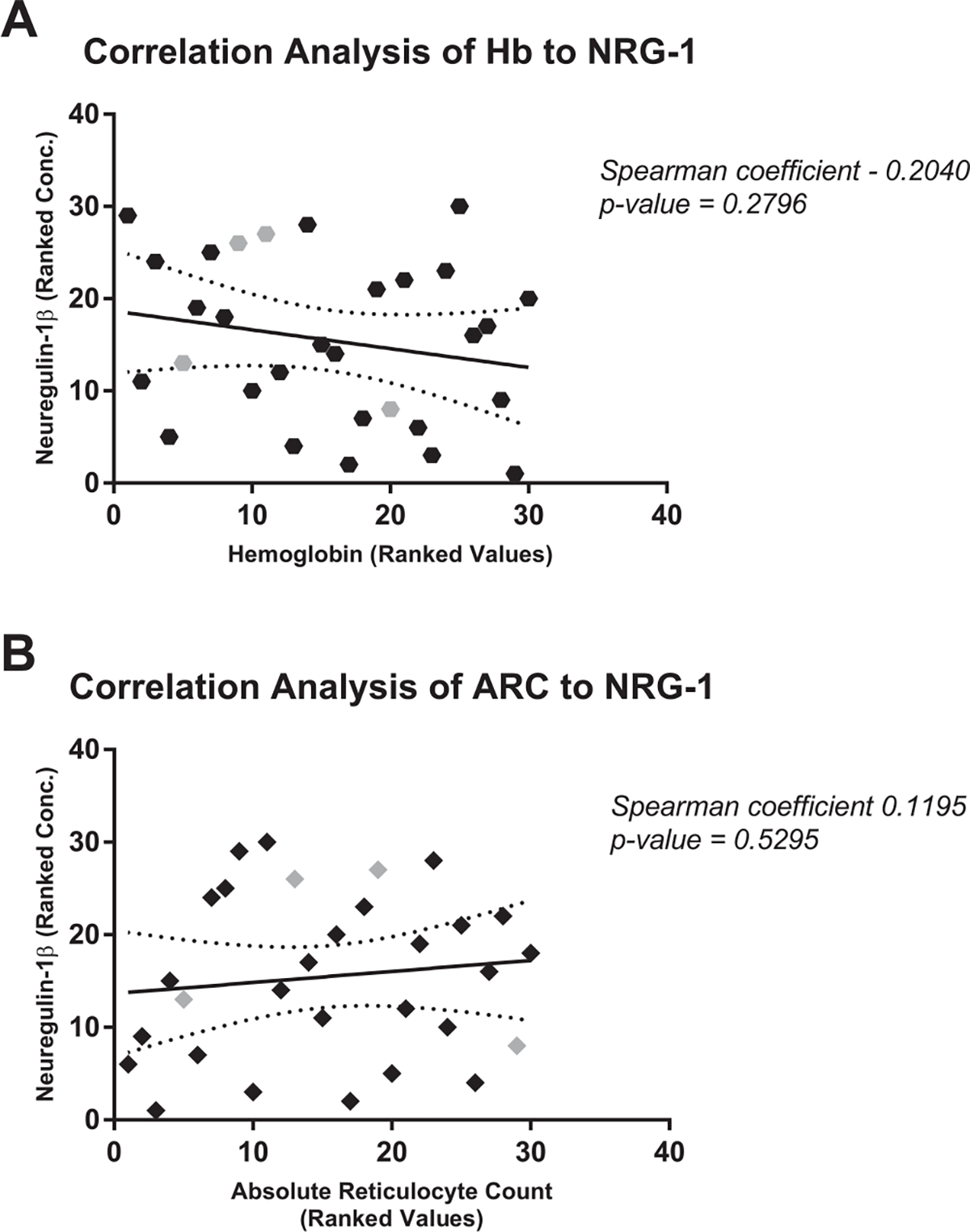
Correlation of Plasma NRG-1 with Hemoglobin and Absolute Reticulocyte Count Plasma NRG-1 levels were not significantly correlated with [A] hemoglobin levels (Hb) or [B] absolute reticulocyte count (ARC). Subjects in gray represent children with SCA who have documented cerebral arteriopathy as defined by cerebral infarct, cerebral arterial stenosis or Transcranial Doppler velocity > 200cm/s. [A] Hexagons represent individuals’ ranked hemoglobin levels correlated to ranked concentrations of plasma NRG-1 including a solid line of best fit and dotted 95% confidence bands. [B] Diamonds represent individuals’ ranked absolute reticulocyte counts correlated to ranked concentrations of plasma NRG-1 including a solid line of best fit and dotted 95% confidence bands.

Neuregulin-1*β* concentrations were not associated with hemoglobin concentration (Spearman r = − 0.2040, p-value = 0.2796) or absolute reticulocytes count in SCA (Spearman r = 0.1195, p-value = 0.5295) (Fig. 2).

## 4. Discussion

Children with SCA had five-fold higher plasma NRG-1 levels in comparison to healthy children, in whom levels were nearly undetectable. Plasma NRG-1 was also positively correlated with elevations in both PDGF-AA and BDNF, previously reported biomarkers of high stroke risk in children with SCA. Because NRG-1 has shown to be protective against cerebral and cerebrovascular injury in a number of models, elevated NRG-1 production in children with SCA may represent an endogenous response to subclinical reduced cerebral ischemia.

Plasma NRG-1 was not associated with total hemoglobin or absolute reticulocyte count, indirect markers of the degree of anemia and hemolysis (Barcellini WaF, 2015, Fehr and Knob, 1979). Of note, the SCA subjects in this study had relatively high levels of Hemoglobin F and were not taking hydroxyurea which likely reflects milder clinical courses. We did not measure other markers of hemolysis, such as LDH, bilirubin, plasma heme and hemoproteins, or soluble VCAM-1, a marker of heme-induced endothelial activation. It is possible that children with SCA who have more severe anemia (and expected higher plasma free heme) may show associations between hematologic values and NRG-1.

Biomarkers of stroke risk, BDNF and PDGF-AA, were elevated in a small number of the SCA subjects, including some WITH cerebral arteriopathy and some without. NRG-1 levels were correlated with both of these biomarkers, suggesting that neuroprotective mechanisms are stimulated in SCA even in the absence of demonstrated neurologic injury or elevated TCD velocities. Together with newer measures of tissue level cerebral flow, such as diffuse correlation spectroscopy, these blood biomarkers may be helpful in identifying children with SCA who have subclinical cerebral ischemia that could affect outcomes such as cognitive functioning (Lee et al., 2019). Blood biomarkers may also be useful adjuncts to stroke risk prediction in resource-limited areas where equipment and staff expertise for TCD ultrasonography are not available.

In non-SCA rodent models it has been demonstrated that elevations in hippocampal NRG-1 and ErbB4 receptor expression peak under acute conditions of cerebral hypoperfusion (Hei et al., 2018). In addition, NRG1 and ErbB4 expression has been shown to increases significantly in acute phases of induced cerebral ischemia and in traumatic brain injury (Tokita et al., 2001, Hei et al., 2018, Erlich et al., 2000). Conversely, it has been shown that reduction of endogenous NRG-1, as observed in NRG-1 +/− mice, results in exacerbated neuronal injury following induced cerebral ischemia (Noll et al., 2019). These studies suggest that ischemic cerebral insults act as driving forces for elevated NRG-1 expression, while reduced NRG-1 expression under similar conditions may be detrimental. Administration of recombinant NRG-1 is currently being tested as a novel therapy for reducing injury in myocardial infarction (ClinicalTrials.gov identifiers NCT01251406; NCT03388593) and soon to be tested in non-SCA stroke (https://news.ucr.edu/articles/2019/05/09/hope-horizon-treatingstroke). This additive approach has proven beneficial in non-SCA animal models of cerebral injury and ischemic stroke, however with the unique internal milieu of SCA, it is imperative to investigate the efficacy of such treatments in a model which recapitulates this complex environment closely. Future studies utilizing the Townes mouse model of SCA may provide clearer insights as to the neuroprotective role of NRG-1 in SCA associated cerebral injury and stroke (Wu et al., 2006). Use of this model may permit mechanistic studies which more closely identify drivers of endogenous NRG-1 expression.

### 4.1. Limitations

Our cohort was relatively small to accommodate study constraints, however the differences identified between groups were statistically significant. While age inclusion criteria were the same for both SCA and Control subjects, younger healthy children and their parents were less likely to consent for blood draw unless they needed it for clinical care. This led to subjects with SCA to be significantly younger than Controls (7.8 ± 3.6 vs 11.4 ± 3.4 years), with resultantly lower weight, stature, and BMI.

Inclusion criteria for SCA subjects in this study included no transfusions in 3 months or hydroxyurea therapy in 6 months. Chronic RBC transfusions and hydroxyurea are disease modifying treatments for those with more severe SCA clinical complications. By excluding individuals with SCA who are receiving these treatments, we eliminated the confounding effects of therapy, but created bias in our cohort for individuals with less severe SCA complications. This is evident by the observed elevated fetal hemoglobin percentage in our children with SCA which is usually associated with lower likelihood of complications related to sickled red blood cells (Urbinati et al., 2015).

Brain MRI screening of all children with SCA for silent cerebral infarction is not part of the clinical practice guidelines in our institution, so this information is incomplete for the SCA subjects. It is possible that subjects with SCA who had silent cerebral infarctions may account for those with higher NRG-1, BDNF or PDGF-AA levels. However, among 190 subjects in the Silent Cerebral Infarct Transfusion Multi-Center Clinical Trial (SIT Trial), there were no significant associations between BDNF levels and the presence or absence of silent cerebral infarcts (Lance et al., 2020). In our cross-sectional study, we are unable to determine the trend of NRG-1, BDNF, PDGF-AA, versus cerebral arteriopathy or progression to stroke over time for these subjects.

A prospective study of a larger group of children with SCA correlating these blood biomarkers with TCD velocities and diffuse correlation spectroscopy would be informative in understanding the relationship of the biomarkers of cerebral perfusion in children with SCA.

### 4.2. Conclusions

This is the first study to document plasma NRG-1 concentrations in children with SCA. While the cohort was small, the differences identified between groups were statistically significant. Our data are intriguing in that some subjects without known cerebral arteriopathy have elevations of neuroprotective proteins, BDNF and NRG-1, suggesting a response to subclinical cerebral ischemia in children with SCA. Together with other blood biomarkers of stroke risk, NRG-1 may be a useful diagnostic test for impaired cerebral perfusion. Further investigation is needed to better understand the mechanistic role of this molecule in brain health and function in SCA.

## Supplementary Material

1

## Figures and Tables

**Table 1:** Subject characteristics for children with SCA and healthy Controls. Subject characterization for both Children with SCA and healthy Control groups. Data are reported as Mean ± Standard Deviation and “n/a” is used where statistical analysis is not applicable.

	Children with SCA	Controls	p-value

n	30	10	n/a
Males/Females	10/20	3/7	n/a
% African American	100	100	n/a
Age (years)	7.8 ± 3.6	11.4 ± 3.5	0.0094
Weight (kg)	26.8 ± 14.7	49.6 ± 16.3	0.0002
Height (cm)	124.8 ± 21.9	151.9 ± 16.3	0.0010
BMI (kg/m^2^)	16.4 ± 2.8	20.7 ± 3.3	0.0002
BMI percentile for age	43.9 ± 28.4	80.6 ± 8.8	0.0003

**Table 2:** Hematological values for children with SCA and healthy Controls. Complete blood count and hemoglobin electrophoresis results including red blood cell count (RBC), hemoglobin (Hb), mean corpuscle volume (MCV), fetal hemoglobin percentage (HbF), white blood cell count (WBC), absolute reticulocyte count (ARC), and platelet count (Plt). White blood cell count, absolute reticulocyte count, and platelet data was available for children with SCA, but not collected in Controls. Normal WBC count ranges from 4.5 to 11.0 × 10^9^ cells/L, normal ARC ranges from 50.0 to 100.0 × 10^9^ cells/L, and normal Plt count ranges from 150.0 to 400.0 × 10^9^ cells/L (Blumenreich, 1990, Priya and RS, 2014, Daly, 2011). Symbol “Ŧ” represents average ranges gathered from medical literature. Values are reported as Mean ± Standard Deviation and “n/a” is used where statistical analysis is not applicable.

	Children with SCA	Controls	p-value

n	30	10	n/a
RBC (10^12^ cells/L)	3.1 ± 0.6	4.5 ± 0.3	<0.0001
Hb (g/dL)	8.8 ± 1.0	12.6 ± 0.6	<0.0001
MCV (fL/cell)	83.2 ± 8.5	85.2 ± 6.0	0.4861
HbF (%)	14.2 ± 7.3	All less than 1.0	< 0.0001
WBC (10^9^ cells/L)	9.9 ± 2.7	^Ŧ^ 4.5–11.0 (Blumenreich, 1990)	n/a
ARC (10^9^ cells/L)	312.3 ± 126.5	^Ŧ^ 0.0–100.0 (Priya and RS, 2014)	n/a
Plt (10^9^ cells/L)	396.4 ± 120.3	^Ŧ^ 150.0– 400.0 (Daly, 2011)	n/a

## Data Availability

The data that support the findings of this study are available from the corresponding author upon reasonable request.

## Abbreviations:

AA: Hemoglobin AA
AC: Hemoglobin AC
ARC: Absolute reticulocyte count
AS: Hemoglobin AS
BDNF: Brain derived neurotrophic factor
BMI: Body mass index
ELISA: Enzyme-linked immunosorbent assay
ErbB: Erythroblastic oncogene B
Hb: Hemoglobin
HbF: Fetal hemoglobin
IQR: Interquartile range
IRB: Institutional review board
LDH: Lactate dehydrogenase
MCAO: Middle cerebral artery occlusion
MCV: Mean corpuscle volume
MRI: Magnetic resonance imaging
MRA: Magnetic resonance angiography
N/A: Not applicable
NRG-1: Neuregulin-1*β*
PDGF-AA: Platelet derived growth factor type-aa
Plt: Platelet
RBC: Red blood cell
RPM: Rotations per minute
SCA: Sickle cell anemia
SCD: Sickle cell disease
SS: Hemoglobin SS
TCD: Transcranial Doppler
VCAM-1: Vascular cell adhesion molecule 1
WBC: White blood cell
