# Supplementary material for: Elevated neuregulin-1β levels correlate with plasma biomarkers of cerebral injury and high stroke risk in children with sickle cell anemia
Source: Endocr Metab Sci. Author manuscript; Available in PMC 2022 Aug 4. (PMC9351492; doi:10.1016/j.endmts.2021.100088)
Supplement: 1 [file NIHMS1816649-supplement-1.pdf]

Peer Review Overview

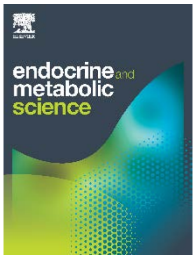

Manuscript Title: Elevated Neuregulin-1β Levels Correlate with Plasma Biomarkers of Cerebral Injury and High Stroke Risk in Children with Sickle Cell Anemia

Received	Jun 29, 2020
1st Decision	Oct 28, 2021
1st Revision Submitted	Nov 27, 2021
Accepted	Feb 10, 2021

1st Decision letter

Reference: ENDMTS-D-20-00039
Title: Elevated Neuregulin-1β Levels Correlate with Plasma Biomarkers of Cerebral Injury and High Stroke Risk in Children with Sickle Cell Anemia
Journal: Endocrine and Metabolic Science

Dear Mr. Chambliss,

Thank you for submitting your manuscript to Endocrine and Metabolic Science.

We have completed the evaluation of your manuscript. The reviewers recommend reconsideration of your manuscript following revision. I invite you to resubmit your manuscript after addressing the comments below. Please resubmit your revised manuscript by Nov 27, 2020.

When revising your manuscript, please consider all issues mentioned in the reviewers' comments carefully: please outline in a cover letter every change made in response to their comments and provide suitable rebuttals for any comments not addressed. Please note that your revised submission may need to be re-reviewed.

Endocrine and Metabolic Science values your contribution and I look forward to receiving your revised manuscript.

Sincerely,

Zeynep Madak-Erdogan

Editor-in-Chief
Endocrine and Metabolic Science

Comments from Editors and Reviewers:

Reviewer #1:

This study from Morehouse School of Medicine investigators led by Christopher Chambliss analyses expression levels of neuregulin-1β (NRG-1) in plasma of children with Sickle Cell anemia (SCA). Thirty children with SCA were compared to ten controls who were African Americans matching by age. Higher levels of NRG-1 were observed, along with previously described higher levels of PDGF-AA and BDNF. Levels of NRG-1 correlated with levels of PDGF-AA and BDNF, which were previously associated with increased stroke in children with SCA. Thus, this study concluded that NRG-1 levels are higher in SCA. This is a well written short report that connects for the first time NRG-1 levels to SCA. What is unclear is whether increased NRG-1 levels are indicative of cerebral ischemia or any pathogenesis associated with SCA. Because many factors are upregulated in SCA, including pro and anti-inflammatory cytokines, factors of vascular and endothelial injury, iron metabolism and so on, further functional analysis is needed to correlate NRG-1 to the underlying pathological process and establish its importance as a biomarker in SCA.

Reviewer #2:

The authors performed a cross-sectional study of children to compare Neuregulin-1β in blood samples of health African American controls (n= 10) and SCA African American children with SCA (n=30). Neuregulin-1β in plasma was measured using enzyme-linked immunosorbent assay. Neuregulin-1β levels were significantly higher in plasma samples of children with SCA when compared with healthy controls. Neuregulin-1β was also positively correlated with BDNF and PDGF-AA concentrations (biomarkers of cerebral injury in children with SCA).

The manuscript is well conceptualized and well written. The strength of the manuscript is the novelty of the topic addressed. The aim to identify new biomarkers to predict or follow the cerebrovascular disease in these children is worth pursuing. However, in its current form, the manuscript reports preliminary data of very limited clinical value. The low number of participants included in the study precludes, for example, a powered analysis of whether Neuregulin-1β levels are higher in SCA children with cerebrovascular when compared to those without cerebrovascular disease.

Other major and minor comments:

1. The introduction is too long.
2. Please write g for centrifuge units instead of RPM.
3. Please clarify "statistical significance was set at *p < 0.05, **p < 0.01, ***p < 0.001 and ****p < 0.0001, at α=0.05".
4. A reasonable attempt to match sex was made. However, there were significantly more older children in the control group, meaning that the two groups are not similar.
5. Methods section does not describe if all SCA children were screened by TCD and MRA to define the presence or absence of "cerebral arteriopathy". Also, TCD and MRA methods should be described in the

Methods section.
6. Were the patients screened for silent infarcts using MRI? If no, this limitation needs to be discussed in depth.
7. Since there is no longitudinal follow-up, it is unclear if higher Neuregulin-1β levels are a result of vasculopathy or a factor contributing to the development of vasculopathy.
8. The discussion did not discuss the clinical value of using Neuregulin-1β in comparison to the TCD screening. What new predictive or pathological information will this biomarker provide to follow these patients?
9. Please provide a conclusion at the end of the discussion section.

Author's 1st Response Letter

Response to comments from Editors and Reviewers:

Reviewer #1:

Reviewer Comment: "What is unclear is whether increased NRG-1 levels are indicative of cerebral ischemia or any pathogenesis associated with SCA. Because many factors are upregulated in SCA, including pro and anti-inflammatory cytokines, factors of vascular and endothelial injury, iron metabolism and so on, further functional analysis is needed to correlate NRG-1 to the underlying pathological process and establish its importance as a biomarker in SCA."

Action: We agree that the manuscript as originally written did not provide supporting information as to the potential underlying pathological processes which may be contributing to the observed elevations in NRG-1 among individuals with SCA. To provide this support we identified a number of additional manuscripts which have employed functional studies to identify factors which contribute to increased endogenous NRG-1 expression both in vivo and in vitro. [See Section 4.0 Discussion, Paragraph 4]

Though many studies have investigated and identified the neuroprotective effects of exogenous NRG-1, few have explored the pathologies which contribute to increased endogenous NRG-1 production. Of those, cerebral injury and ischemia have been identified as two potential drivers. Our study is novel in that it identifies sickle cell anemia as a disease in which endogenous NRG-1 is significantly elevated and correlated to other biomarkers of cerebral ischemia and stroke risk. It is beyond the scope of the current work to determine cause of elevated NRG-1 levels, but this observation raises important questions which can be further expounded upon. We updated the manuscript to highlight this need by suggesting future studies to further pinpoint exact pathologies contributing to increased NRG-1 production in SCA. [See Section 4 Discussion, Paragraph 5] Detailed Response to Reviewers Additional updates to the manuscript in response to reviewer's suggestions can be seen throughout the Discussion section.

Reviewer #2:

Reviewer Comment: "… the manuscript reports preliminary data of very limited clinical value. The low number of participants included in the study precludes, for example, a powered analysis of whether Neuregulin-1β levels are higher in SCA children with cerebrovascular when compared to those without cerebrovascular disease."

Action: We acknowledge that our sample size was small. Due to the widespread use of hydroxyurea and TCD screening to identify children at risk for stroke, the number of children untreated with hydroxyrea or transfusions eligible to participate during the study period was limited. However, even with the small number of subjects, we identified statistically significant differences in NRG-1 levels compared to healthy Controls, and that all three biomarkers were elevated (above third quartile) even in some children without cerebral arteriopathy. This is a novel finding that suggests the possibility of cerebral ischemia even in those children with SCA who don't have abnormal TCD or brain MRA findings. The discussion has been revised to include this [Section 4 Discussion, paragraph 3]

Reviewer Comment: "The introduction is too long"
Action: The introduction has been made more concise and shortened significantly (removed ~100 words) while retaining relevant information. [See Section 1 Introduction]

Reviewer Comment: "Please write g for centrifuge units instead of RPM."
Action: Updated centrifuge units to g using formula RCF (g) = 1.12*Radius*((RPM/1000) ^2; Given Radius = 186mm, RCF (g) = 2552 [See Section 2.4 Immunoassays, Paragraph 1]

Reviewer Comment: "Please clarify "statistical significance was set at *p < 0.05, **p < 0.01, ***p < 0.001 and ****p < 0.0001, at α=0.05""
Action: Threshold for statistical significance and the associated verbiage was clarified in the Methods [See Section 2.5 Statistical Analysis]

Reviewer Comment: "A reasonable attempt to match sex was made. However, there were significantly more older children in the control group, meaning that the two groups are not similar."
Action: Differences were identified in the Results and discussed further in Limitations [See Section 3.1 Subject Characteristics; Section 4.1 Limitations, Paragraph 1]

Reviewer Comment: "Methods section does not describe if all SCA children were screened by TCD and MRA to define the presence or absence of "cerebral arteriopathy". Also, TCD and MRA methods should be described in the Methods section."
Action: Methods section updated to include this information [See Section 2.2 Neuroimaging]

Reviewer Comment: "Were the patients screened for silent infarcts using MRI? If no, this limitation needs to be discussed in depth."
Action: This information was added to Methods section and further discussed in the limitations [See Section 2.2 Neuroimaging; Section 4.1 Limitations, Paragraph 3]

Reviewer Comment: "Since there is no longitudinal follow-up, it is unclear if higher Neuregulin-1β levels are a result of vasculopathy or a factor contributing to the development of vasculopathy."
Actions: Limitations of cross-sectional study design along with benefits of including longitudinal follow-up are added to the Discussion [See Section 4.1 Limitations, Paragraph 4]

Reviewer Comment: "The discussion did not discuss the clinical value of using Neuregulin-1β in comparison to the TCD screening. What new predictive or pathological information will this biomarker provide to follow these patients?"
Action: This utility added to the Discussion [See Section 4 Discussion, Paragraph 3]

Reviewer Comment: "Please provide a conclusion at the end of the discussion section."
Action: Conclusion now added to the Discussion [See Section 4.2 Conclusion] Additional updates to the manuscript in response to reviewer's suggestions can be seen throughout the Methods and Discussion sections.

Accept letter

Reference: ENDMTS-D-20-00039
Title: Elevated Neuregulin-1β Levels Correlate with Plasma Biomarkers of Cerebral Injury and High Stroke Risk in Children with Sickle Cell Anemia
Journal: Endocrine and Metabolic Science

Dear Mr. Chambliss,

Thank you for submitting your manuscript to Endocrine and Metabolic Science.

We are pleased to inform you that your manuscript has been accepted for publication.

My comments, and any reviewer comments, are below.

Your accepted manuscript will now be transferred to our production department. We will create a proof which you will be asked to check, and you will also be asked to complete a number of online forms required for publication. If we need additional information from you during the production process, we will contact you directly.

We appreciate you submitting your manuscript to Endocrine and Metabolic Science and hope you will consider us again for future submissions.

Sincerely,

Zeynep Madak-Erdogan

Editor-in-Chief
Endocrine and Metabolic Science

Editor and Reviewer comments:

Reviewer #1: No more comments

Reviewer #2: The authors have satisfactorily answered all of the comments and suggestions made by this reviewer. I believe the manuscript is suitable for publication.

-------- End of Review Comments --------
